# Preparation of SnS_2 _colloidal quantum dots and their application in organic/inorganic hybrid solar cells

**DOI:** 10.1186/1556-276X-6-298

**Published:** 2011-04-05

**Authors:** Furui Tan, Shengchun Qu, Ju Wu, Kong Liu, Shuyun Zhou, Zhanguo Wang

**Affiliations:** 1 Key Laboratory of Semiconductor Materials Science, Institute of Semiconductors, Chinese Academy of Sciences, P.O. Box 912, Beijing 100083, PR China; 2 Key Laboratory of Photochemical Conversion and Optoelectronic Materials, TIPC, Chinese Academy of Sciences, Beijing 100191, PR China

## Abstract

Dispersive SnS_2 _colloidal quantum dots have been synthesized via hot-injection method. Hybrid photovoltaic devices based on blends of a conjugated polymer poly[2-methoxy-5-(3",7"dimethyloctyloxy)-1,4-phenylenevinylene] (MDMO-PPV) as electron donor and crystalline SnS_2 _quantum dots as electron acceptor have been studied. Photoluminescence measurement has been performed to study the surfactant effect on the excitons splitting process. The photocurrent of solar cells with the hybrid depends greatly on the ligands exchange as well as the device heat treatment. AFM characterization has demonstrated morphology changes happening upon surfactant replacement and annealing, which can explain the performance variation of hybrid solar cells.

## Introduction

Organic-inorganic hybrid bulk-heterojunction (BHJ) solar cell is an interesting alternative to the all-organic solar cells with replacement of the organic n-type material with inorganic nano-particles (NPs). This new kind of solar cell aims at combining the solution processability of conjugated polymers with high electron mobility and the relative environmental stability of inorganic semiconductors. Up to now, various NPs such as CdSe [1-3], PbS [4], TiO_2 _[5], ZnO [6], or heterojunction nano-crystals [7-9] have been applied in organic-inorganic BHJ solar cells. The best solar cell adopting inorganic nano-phase as the electron acceptor demonstrated a power conversion efficiency exceeding 3% using CdSe tetrapods [3]. However, compared with this toxic material, a nontoxic, environmental-friendly alternative may be more attractive in the future application of this kind of cells.

Tin disulfide (SnS_2_) is a fullerene-like semiconductor with a band gap of about 2.35 eV [10]. In this study, the consideration of SnS_2 _as electron acceptor in BHJ solar cell is based on its several advantages. Comparing with Cd-containing inorganic nanoparticles, SnS_2 _is easy to prepare, nontoxic, and environment-friendly, with an abundant content of row materials in the earth. Besides, it has an appropriate energy level distribution when forming hybrid with an electron donor such as MDMO-PPV; electrons transfer at the interface is convenient. Furthermore, as a fullerene-like semiconductor, it is easy to form a net-like interpenetrating connection between particles, which is greatly beneficial to electrons transportation. Up to now, extensive research has been focused on its application of gas-sensoring [11], lithium batteries [12], and electrical switching [13]. As a photoconductive semiconductor, its potential application in solar cells is rear. Our previous study has shown that SnS_2 _nano-particles with a crystalline/amorphous blended phase demonstrated obvious photovoltaic property as an electron acceptor when blending with an organic semiconductor poly[2-methoxy-5-(3",7"-dimethyloctyloxy)-1,4-phenylenevinylene] (MDMO-PPV) [14]. However, the conversion efficiency may be influenced due to the not well-dispersed and well-crystallized SnS_2 _particles prepared at low temperature. Other commonly used methods such as solvothermal [15] or chemical and vapor deposition [16] usually generated very large particles, which is not suitable for hybrid BHJ solar cells. A dispersive and well-crystallized SnS_2 _nano-particle is of great necessity to form an interpenetrating electron tunneling path in the hybrid BHJ solar cells.

Herein, SnS_2 _colloidal quantum dot that is up to the role in organic/inorganic hybrid BHJ solar cell is prepared via hot-injection method [17,18]. The hybrid solution containing SnS_2 _quantum dots and MDMO-PPV in chloroform or chlorobenzene is clear and transparency. Solar cells using these quantum dots as electron acceptor generate an improved photovoltaic property after replacing the insulating surfactant on the particles surface. The result of this research suggests this easy-prepared nontoxic semiconductor could be a promising candidate for BHJ solar cells.

## Experiment

### Synthesis of SnS_2 _colloidal quantum dots via hot-injection method

All chemicals are used as-received without further treatment. In a typical reaction, 0.26 g of tin (IV) chloride anhydrous (SnCl_4_, 99.0+%, Sinopharm, Beijing, China) is added to 20 ml of oleylamine (OLA, 80+%, Aladdin, Shanghai) in a 50-ml three-neck flask. The mixture is purged with N_2 _for 30 min at 120°C and heated to 200°C. Then 3 ml of OLA containing 0.22 g of thioacetamide (TAA, 99.0+%, Sinopharm, Beijing, China) is injected into the mixture quickly. The reaction is kept at 200°C with N_2 _purging for 12 h and then cooled down to room temperature. The as-formed nanocrystals are isolated by precipitation with ethanol followed by centrifugation. The final product, SnS_2 _quantum dot, is washed three more times by solvent/antisolvent precipitation with chlorobenzene/ethanol.

For a better application in hybrid BHJ solar cell, the OLA ligands on the surface of SnS_2 _colloidal quantum dots are partly replaced by stirring the final product in anhydrous pyridine at 60°C for 1 h and ultrasonicated at 40°C for 1 h. After that, the particles are precipitated with hexanes at room temperature, recollected by centrifugation, and then dispersed into a mixture of chlorobenzene/pyridine (90:10, vol/vol) for further use.

### Fabrication of hybrid bulk-heterojunction solar cells

The fabrication process of hybrid BHJ solar cells is as follows. Poly(thiophene) (3,4-ethylenedioxythiophene)/poly(styrenesulfonate) (PEDOT:PSS) is spin coated at 2400 rpm onto the ITO substrates those are precleaned by soap water followed by deionized water, and then ultrasonicated in acetone and isopropanol. After the ITO/PEDOT:PSS is annealed at 140°C for 1 h, it is transferred into a glove box together with the organic/inorganic-blended solution. The mixture is prepared by blending SnS_2 _and MDMO-PPV in chlorobenzene with different mass ratios and then ultrasonicated for about 1 min to form a transparent solution. Hybrid BHJ films with an optimized thickness of about 120 nm are achieved by spin-coating the mixture on PEDOT:PSS at 1500 rpm for 30 s in a N_2 _atmosphere in a glove box. Afterward, onto the hybrid film, a ZnO buffer layer of about 20 nm is obtained by spin-coating a ZnO methanol solution (30 mg/ml, 3000 rpm) [19]. The solar cells fabrication is then finished by thermally depositing a 100-nm aluminum cathode on top.

The crystalline phase pattern of SnS_2 _particles is characterized by X-ray diffraction (XRD) on a Rigaku D/max-gA X-ray diffractometer with Cu Kα radiation. Its morphology is given by a transmission electron microscopy (TEM) on a Hitachi H-800 at an acceleration voltage at 80 kV. Absorption spectrum (Abs) and photoluminescence (PL) measurements are carried out on Varian U-3000 model ultraviolet-visible spectrophotometer and Varian Cary Eclipse fluorescence spectrophotometer, respectively. The surface morphology of hybrid MDMO-PPV:SnS_2 _films is characterized on Solver P47 scanning probe microscopy (SPM). The current-voltage (*I*-*V*) measurements on the MDMO-PPV:SnS_2 _BHJ solar cells are performed on Keithley 2400 source in forward bias mode under AM 1.5 100 mW/cm^2 ^illumination.

## Results and discussion

### Characterization of SnS_2 _and its hybrid with MDMO-PPV

**Figure 1:**
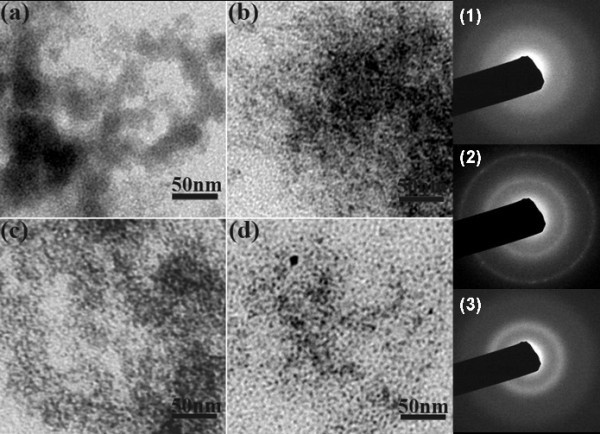
**TEM images of SnS_2 _particles reacted for different times**. **(a) **0.25 h, **(b) **5 h, **(c) **12 h, and **(d) **30 h. Scale bars in the images are 50 nm.

**Figure 2:**
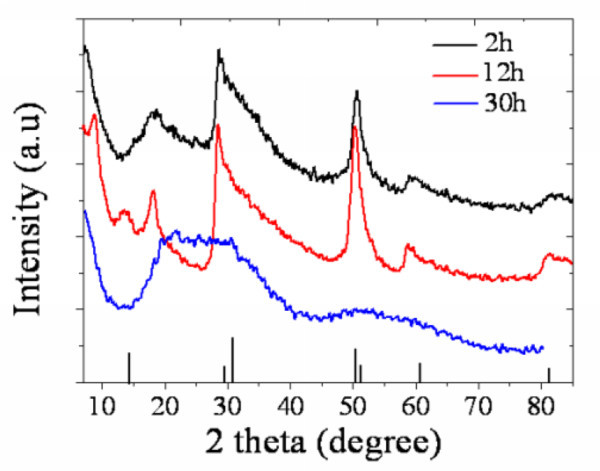
**XRD pattern of SnS_2 _particles at different reaction times**.

The TEM images and SEAD measurement of SnS_2 _nano-particles distilled at different reaction times are shown in Figure 1. Corresponding to morphology in Figure 1a, SEAD in Figure 1 (1) exhibits an amorphous phase and the beginning reaction. It turns to a formation of polycrystalline when further increasing reaction time, and then again, amorphization formed. It reveals such a reaction process as precursor decomposition (Figure 1a) followed by quantum dot precipitation (Figure 1b,c) and then redissolving into the solution (Figure 1d). In fact, after the TAA-OLA solution was injected, a phenomenon was observed that the reaction mixture first turned from turbid orange to semi-transparent yellow and then becomes clearly transparent yellow. When the reaction temperature was increased, just less time was needed for this variation. It can be seen from Figure 1 that an optimal reaction for SnS_2 _colloidal quantum dots was happened at 12 h, which generated a dispersive SnS_2 _quantum dot about 5-7 nm in size. The crystalline phase of SnS_2 _particles is demonstrated by XRD in Figure 2 from which the crystalline transformation process can also be observed. All the characteristic diffraction peaks corresponding to a berndtite-4 type (PDF card 21-1231) appear when the reaction persists for 12 h. Nano-size of SnS_2 _at this time is about 5.3 nm, which is obtained from the Scherrer equation *D *= *K λ*/*β *cos *θ *where *D *is the diameter of the synthesized crystals, *β *is the full-width half-maximum, and *θ *is the diffraction angle. Further reaction up to 30 h caused dissolving of SnS_2 _particles through forming coordinated organic compound with OLA. Thus, diffraction signal mainly demonstrate an amorphous phase in the longer time reacted product.

**Figure 3:**
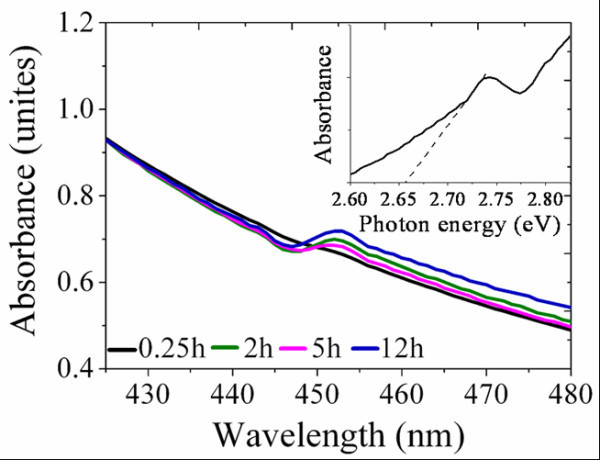
**Normalized light absorption of SnS_2 _reacted for different times**. The inset gives the 12-h sample's absorption property in form of photon energy.

**Figure 4:**
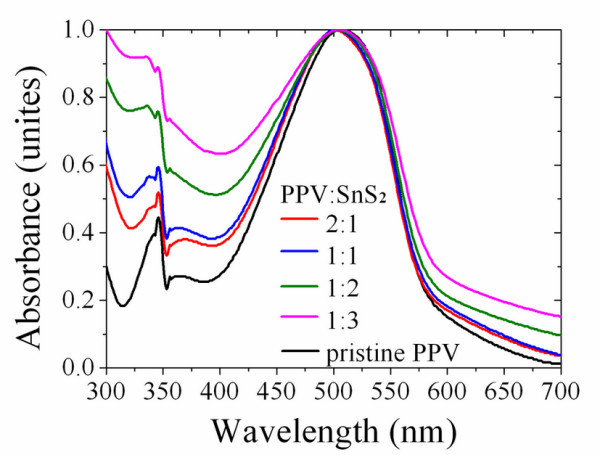
**Normalized light absorption of MDMO-PPV and MDMO-PPV:SnS_2 _hybrid materials with different weight ratios**.

The absorption spectra of SnS_2 _nano-particles in chlorobenzene are shown in Figure 3. SnS_2 _sample reacted for 0.25 h does not show an obvious absorption near its bandgap energy region. It is due to the presence of an amorphous precursor that is not crystallized. Samples at 2 and 5 h exhibit obvious absorption peaks at 450 nm, suggesting the formation of nano-particles. The absorption of SnS_2 _with a final reaction time of 12 h is a little intensified and shows a slight red-shift, which is caused by a enlarged particle size. From the absorption spectrum, the optical bandgap of SnS_2 _particles can be obtained which is shown as the inset in Figure 3. SnS_2 _nano-particles reacting for 12 h generated a band-gap value of about 2.66, 0.3 eV larger than its bulk phase due to the quantum size effect. The absorption properties of SnS_2 _and MDMO-PPV blends in chlorobenzene are given in Figure 4. It is simply a superposition of the respective absorption spectra of pure MDMO-PPV and SnS_2 _nano-particles at longer and shorter wavelength. The absorption of SnS_2 _at 450 nm in the blends is not obvious; this is probably due to its weaker absorbance comparing to MDMO-PPV. The introduction and increasing weight ratio of SnS_2 _in organic/inorganic hybrids cause more obvious absorption enhancement in ultra-violet region, mainly because of the intensive absorption property of nano-particles [20-22]. Although light absorption enhancement is commonly existed in organic-inorganic hybrid systems due to the introduction of inorganic nano-particles, its contribution to the photocurrent of hybrid solar cells is not confirmable because of many other caused variations such as excitons splitting and so on.

**Figure 5:**
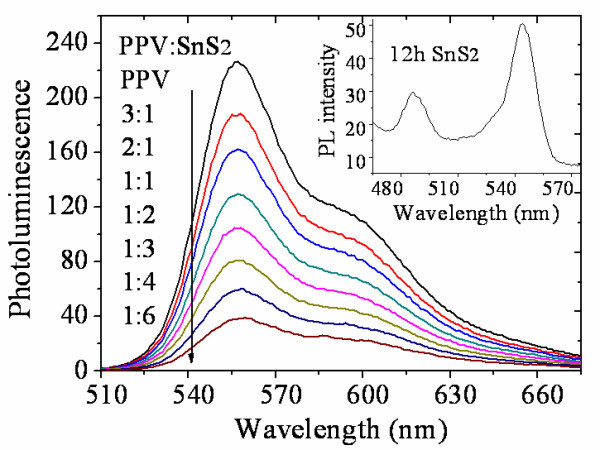
**Photoluminescence spectrum of SnS_2 _and MDMO-PPV blends with different SnS_2 _concentration**. The inset shows the PL spectrum of SnS_2 _particle reacted for 12 h.

Photoluminescence (PL) spectrum is used to research how the addition of SnS_2 _affects the photo-generated charge transfer in the hybrid. Figure 5 shows the PL quenching of MDMO-PPV following the increasing concentration of SnS_2_. Indeed, the spectra show that the PL intensity is increasingly lost upon addition of SnS_2 _due to the fast, sub-picosecond forward electron transfer from MDMO-PPV to SnS_2_. At 50 wt% SnS_2_, about 40% of the PL is quenched, and at 90 wt% SnS_2_, 80% of the PL is quenched. Further increasing of the concentration of SnS_2 _will not obviously quench the PL. This is attributed to a saturated organic/inorganic interface, which will further decrease the lifetime of photo-generated excitons [23]. Inset in Figure 5 shows the PL spectrum of SnS_2 _reacted for 12 h. It exhibits two strong emission peaks at 545 and 490 nm. The former is corresponding to radiative recombination of quantum confined electron-hole pair that in energy is a little smaller than the energy bandgap of nanocrystals [24]. The latter is not clear for us at present. We suppose it may be due to radiated recombination of excitons' absorption-generated electrons lying at higher excited energy levels.

**Figure 6:**
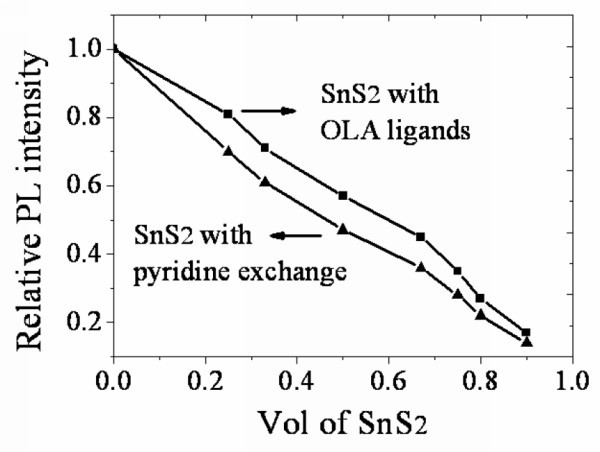
**Relative PL intensity variation of MDMO-PPV:SnS_2 _hybrid before and after pyridine exchange at different SnS_2 _concentrations**.

The surfactant on the SnS_2 _particles affects the electron transfer from MDMO-PPV to SnS_2 _so that the PL quenching intensity may be influenced. This can be demonstrated by replacing the long insulating OLA ligands with pyridine. As is shown in Figure 6, comparing with that of SnS_2 _with OLA ligands on surface, the PL intensity is obviously further decreased after treating with pyridine, suggesting a more efficient charge transfer between the organic and the inorganic materials. This is of great importance for the use of active layers in hybrid solar cells.

### Performance of BHJ solar cells with MDMO-PPV:SnS_2 _hybrid

**Figure 7:**
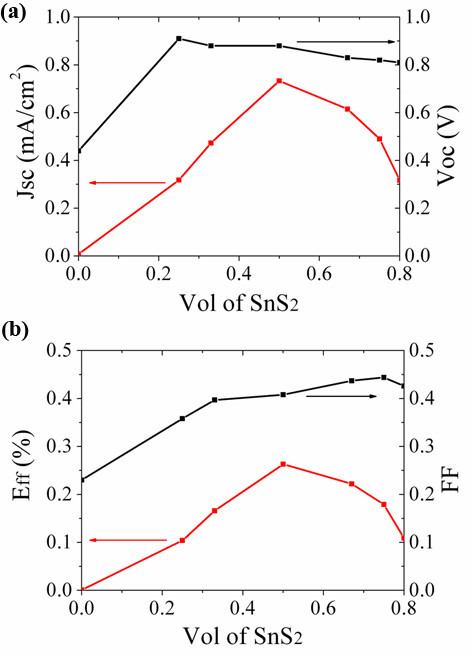
**Photovoltaic performance of MDMO-PPV:SnS**_**2 **_**hybrid solar cells**. **(a) **Jsc (red) and Voc (black) versus wt% SnS_2_. **(b) **FF (black) and Eff (red) versus wt% SnS_2_. SnS_2 _particles in these solar cells were treated with pyridine before use.

Detailed influence of pyridine-processed SnS_2 _concentration in the hybrid film on the photovoltaic performance is given in Figure 7 in which several characteristics can be noted. Figure 7a shows that current density (Jsc) initially increases with increasing amount of SnS_2_, reaches the maximum at 50 wt% SnS_2_, and then decreases with further increasing the SnS_2 _concentration. This trend might be related to the increased formation of free charges inferred from the PL quenching and/or the formation of more percolation pathways of SnS_2 _particles that facilitate electrons transport at initial amount increasing of SnS_2 _[25]. On the other hand, high SnS_2 _concentrations may induce large-scale phase separation due to particles aggregation [26], so that the donor-acceptor contact is affected, which is deleterious to device photocurrent. The open circuit voltage (Voc) decreases slightly when the amount of SnS_2 _increases. This might be due to the formation of shunts that may conduct charges from electrode to electrode. This likelihood increases once increasing the SnS_2 _concentration. Generally, the value of fill factor (FF) is enlarged from 0.23 to 0.45 by increasing the amount of SnS_2 _in the blend. This trend can be analyzed by reasoning that FF is a measure for the balance between free electrons and holes transport. While keeping the polymer concentration unchanged, increasing the SnS_2 _concentration will increase the electrons transport efficiency so that more balanced charge transportation is achieved. The overall trend, when all parameters are combined into the transversion efficiency (Eff) is that it reaches the largest of about 0.26% at 50 wt% SnS_2 _concentration. This concentration is different from the optimal amounts required for ZnO in MDMO-PPV:ZnO blends (67%) [6] and CdSe in PCPDTBT:CdSe blends (90%) [3]. From the curve shape, we can see that the efficiency variation is derived mostly by photocurrent.

**Figure 8:**
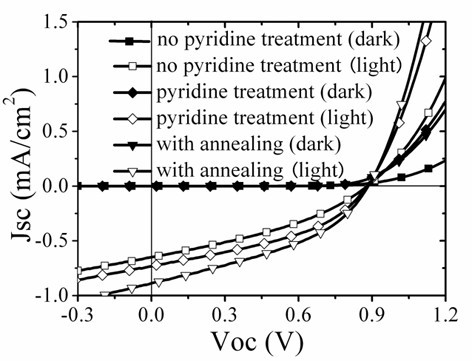
**Dark and light performance of solar cells with SnS_2 _particles before pyridine treatment (square), after pyridine treatment (diamond), and device after annealing (triangle)**. The hybrid active layer contains 50 wt% of SnS_2_.

**Figure 9:**
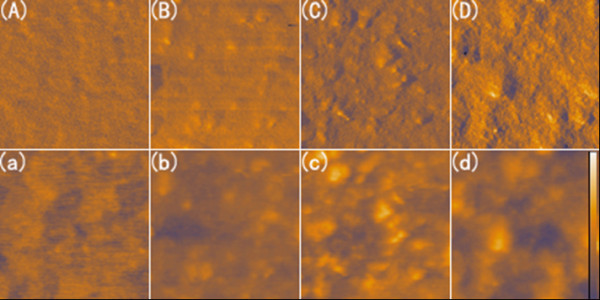
**AFM images of hybrid films (1 μm × 1 μm) with 50 wt% SnS_2_. **(A) phase image of referenced MDMO-PPV film, (B) phase image of hybrid film with as-synthesized SnS_2 _particles, (C) phase image of hybrid film containing pyridine treated SnS_2 _particles, and (D) phase image of MDMO-PPV:pyridine-SnS_2 _film after annealing. (a)-(d) are the corresponding heitht images of (A)-(D). The scale bar in the height image indicates 40 nm.

To study the effect of surfactant on the photovoltaic performance, solar cells with and without pyridine treatment are fabricated and characterized. Also researched is the MDMO-PPV:pyridine-SnS_2 _solar cell annealed at an optimized temperature of 150°C. The performances of solar cells with the same SnS_2 _weight ratio of 50% in the three devices are shown in Figure 8. All the solar cells exhibit good diode behaviors in dark. Compared with the cell without pyridine treating, solar cell with pyridine exchange shows enhanced short circuit current density from 0.65 to 0.73 mA/cm^2 ^while keeping the open circuit voltage and fill factor nearly the same, about 0.88 V and 0.4, respectively. The Jsc exhibits further enhancement up to 0.88 mA/cm^2 ^through annealing. The opti-electric transformation efficiency of solar cell with pyridine is 0.263%, larger than that without pyridine, 0.204%, and this parameter goes up to 0.31% when the device with pyridine was annealed. The increasing in photocurrent upon surfactant exchange can be explained that charge transfer process at the interface of MDMO-PPV and SnS_2 _could be more suited to happen, followed by a convenient free electrons transport among SnS_2 _particles because of the benzene ring and small size of the pyridine molecule. However, an additional dominant reason that induces the Jsc enhancement after annealing might be optimized organic and inorganic phase separation as well as continuous electron transport through compact SnS_2 _connections after the remain solvent's elimination. This can be demonstrated by characterizing the surface morphology of different hybrid films, which are shown in Figure 9.

Compared with the homogeneous and flat MDMO-PPV film as shown in Figure 9A and a, the hybrid film containing OLA-linked SnS_2 _shows clearly two phases (Figure 9B). The SnS_2 _phase that is present as small protrusions (no higher than 20 nm shown in Figure 9b) are well dispersed and immersed into the homogeneous organic phase, which demonstrates that the OLA-linked SnS_2 _particles can form good contact with MDMO-PPV. The SnS_2 _particles show more enlarged aggregation and protrusion after pyridine treating, so that phase separation as caused is clearly observed (Figure 9C). The hybrid film surface becomes rougher due to large SnS_2 _aggregation (Figure 9c). This will cause decreased interface between SnS_2 _and PPV. However, Jsc characteristic of solar cells with pyridine-SnS_2 _as acceptor is superior to that of solar cells with OLA-SnS_2_. One can get the supposition that the excitons splitting efficiency should be compensated upon ligand exchange so that it will not be weakened due to phase separation and interface decreasing. The supposition can be demonstrated through photoluminescence property in Figure 5b that PL quenching happens more intensively than that without pyridine treatment. On the other hand, transportation process of free charges, especially electrons, may be favored from suitable phase separation due to enlarged and connected inorganic phase. After heat treatment, previous small SnS_2 _aggregates, formed during pyridine exchange, will further connect and partly fuse with the adjacent ones (Figure 9D). Thus, electrons can find a more convenient way to transport themselves to the electrode through interpenetrating networks. Besides, the film surface becomes much flatter after annealing (Figure 9d), which is beneficial to form a good contact between the active layer and the electrode. This is why the cell performance has an improvement after annealing. Noticed is that, the efficiency of our solar cell using SnS2 as the electron acceptor is relatively low comparing with other hybrid solar cells such as CdSe [3] and PbS [4]. This is mainly attributed to un-optimized particle morphology as well as the particle aggregation in the hybrid film, increasing the series resistance and decreasing current. On the other hand, SnS_2 _particles enhanced light absorption in the hybrid film is not obvious. Maybe this is our further study to improve the device performance in next step.

## Conclusions

Dispersive SnS_2 _colloidal quantum dots are synthesized and considered as electrons acceptor in hybrid hetero-junction solar cells containing a conjugated polymer (MDMO-PPV). It shows a best performance at the SnS_2 _weight ratio of 50%. OLA ligand on the particles should have an influence on the charges separation in the blends when characterizing through photoluminescence. Surfactant exchange using pyridine causes increased PL quenching, which suggests the enhanced excitons splitting efficiency. Thus, an obvious improvement in photocurrent as well as energy transversion efficiency is realized. Annealing treatment of the devices produces further increase in efficiency due to the enhancement of photocurrent. AFM studies have provided the insights into the variation of devices performance. Comparing with the uniformly distributed two phases in the OLA-SnS_2_:MDMO-PPV blended film, a phase separation in pyridine-SnS_2_:MDMO-PPV blending is appreciated on the photocurrent increase due to the pyridine ligand. Besides, not the isolated aggregates but the connected SnS_2 _networks after annealing are the best for the solar cells performance.

## Abbreviations

Abs: absorption spectrum; BHJ: bulk-heterojunction; SnS_2_: disulfide; NPs: nano-particles; PL: photoluminescence; TEM: transmission electron microscopy; XRD: X-ray diffraction.

## Competing interests

The authors declare that they have no competing interests.

## Authors' contributions

FT participated in the design of the study, carried out the total experiment, performed the statistical analysis as well as drafted the manuscript. SQ participated in the guidance of experiment. JW and SL helped to give the corrections of the manuscript. ZW helped to give the theoretical guidance of the experiment. KL gave some help on the obtaining of the reading papers. All authors read and approved the final manuscript.
